# Supplementary material for: Environmental conditions influencing the abundance of the salmonid ectoparasite Salmincola californiensis across upper Willamette River Reservoirs, Oregon
Source: PeerJ. 2025 Apr 7;13:e19228. doi: 10.7717/peerj.19228 (PMC11984478; doi:10.7717/peerj.19228)
Supplement: Supplemental Information 1 — Photos of a light trap depicting (counterclockwise from top left) the funnel and trap entrance, the LED light used to attract Salmincola californiensis copepodids, and the outside body of the trap with rope harness for attaching to an anchored line. Photos C.A. Murphy. [file peerj-13-19228-s001.docx]

**Appendix A and Supplemental Figure 1**

to accompany

**Environmental conditions influencing the abundance of the Salmonid Ectoparasite Salmincola californiensis across upper Willamette River Reservoirs, Oregon**

Kelsi Antonelli^1^, Christina A. Murphy^2^, Amanda M.M. Pollock^1^, Ivan Arismendi^1^

^1^ Department of Fisheries, Wildlife, and Conservation Sciences, Oregon State University, Corvallis, Oregon, USA

^2^ U.S. Geological Survey, Maine Cooperative Fish and Wildlife Research Unit, Orono, Maine, USA

Corresponding Author:

Christina Murphy^2^

5755 Nutting Hall, Orono, Maine, 04469, USA

Email address: [christina.murphy@maine.edu](mailto:christina.murphy@maine.edu)

**Appendix A. Covariate types, definitions, and descriptions of covariates tested**

| Variable Type | Definition | Covariate | Covariate Format |
| --- | --- | --- | --- |
| Response Variables |  | Copepodid detection and counts in light traps | Detection history, binary (1=detection, 0=not detected)  Counts (continuous) |
| Site-level covariates  (*siteCovs*) | Site = individual trap  (Covariates that change between traps only) | Reservoir  Trap Line | Factor – Cougar, Lookout Point, Fall Creek  Factor |
| Season-level covariates  (*yearlySiteCovs*) | Season = month  (Covariates that change between months) | Trap depth  Water temperature - at trap  Water clarity  Light  Thermocline – trap above or below  Moon  Outflow | Numerical (continuous)  Numerical (continuous) –smoothed curve of temp profile  Numerical (continuous) – light extinction coefficient  Numerical (continuous) – surface light proportion  Binary (0=above, 1=below)  Numerical (continuous) - % fullness  Numerical (continuous) – at dam |
| Observation-level covariates  (*obsCovs*) | Observation event = sampling event  (Covariates that change between sampling events) | Funnel size  Light on/off  *Leptodora* count  Fish count  Fish presence/absence  Total zooplankton abundance  Zooplankton abundance by broad taxa  Copepodid removal | Binary (1=75mm, 0=60mm)  Binary (1=on, 0=off)  Numerical (continuous) – Leptodora abundance in trap  Numerical (continuous) – sculpin abundance in trap  Sculpin in trap, binary (1=present, 0=absent)  Numerical (continuous)  Numerical (continuous)  Binary (1=removed during sample 1, 0=not removed) |


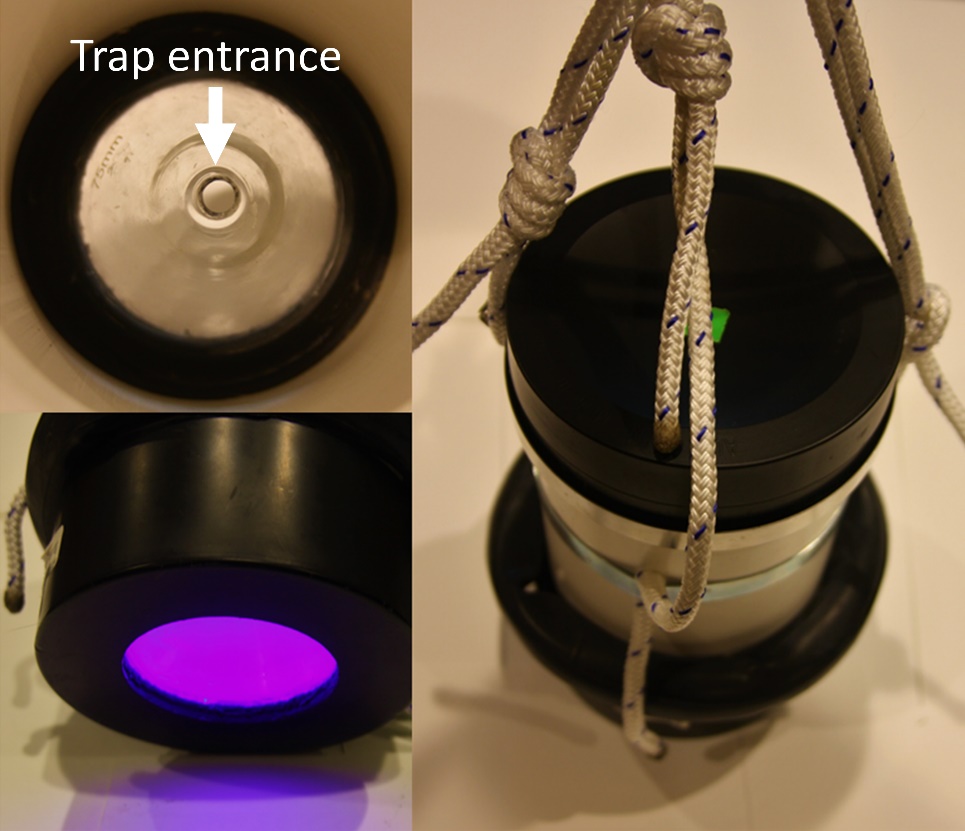


Supplemental Figure 1. Photos of a light trap depicting (counterclockwise from top left) the funnel and trap entrance, the LED light used to attract *Salmincola californiensis* copepodids, and the outside body of the trap with rope harness for attaching to an anchored line. Photos C.A. Murphy.
